# Therapies Targeted at Non-Coding RNAs in Prevention and Limitation of Myocardial Infarction and Subsequent Cardiac Remodeling—Current Experience and Perspectives

**DOI:** 10.3390/ijms22115718

**Published:** 2021-05-27

**Authors:** Michal Kowara, Sonia Borodzicz-Jazdzyk, Karolina Rybak, Maciej Kubik, Agnieszka Cudnoch-Jedrzejewska

**Affiliations:** Chair and Department of Experimental and Clinical Physiology, Laboratory of Centre for Preclinical Research, Medical University of Warsaw, Banacha 1b, 02-097 Warsaw, Poland; michal.kowara@wum.edu.pl (M.K.); sonia.borodzicz@onet.pl (S.B.-J.); karolinamariarybak@gmail.com (K.R.); maciej.kubik1998@gmail.com (M.K.)

**Keywords:** ncRNA, miRNA, lncRNA, circRNA, atherosclerotic plaque, myocardial infarction, cardiac remodeling, cell death

## Abstract

Myocardial infarction is one of the major causes of mortality worldwide and is a main cause of heart failure. This disease appears as a final point of atherosclerotic plaque progression, destabilization, and rupture. As a consequence of cardiomyocytes death during the infarction, the heart undergoes unfavorable cardiac remodeling, which results in its failure. Therefore, therapies aimed to limit the processes of atherosclerotic plaque progression, cardiac damage during the infarction, and subsequent remodeling are urgently warranted. A hopeful therapeutic option for the future medicine is targeting and regulating non-coding RNA (ncRNA), like microRNA, circular RNA (circRNA), or long non-coding RNA (lncRNA). In this review, the approaches targeted at ncRNAs participating in the aforementioned pathophysiological processes involved in myocardial infarction and their outcomes in preclinical studies have been concisely presented.

## 1. Introduction

**Figure 1:**
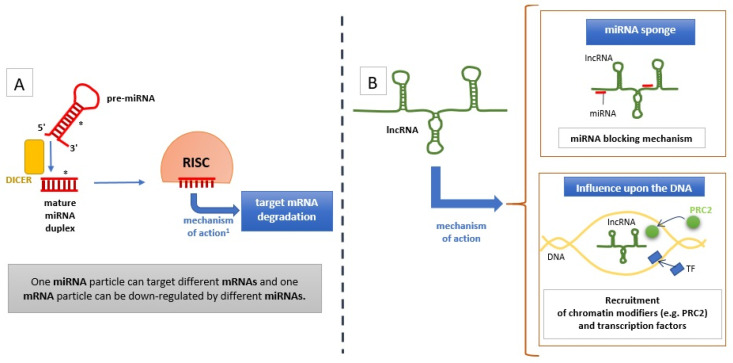
Mechanism of action of miRNA (**A**) and lncRNA (**B**). * = miRNA fragment more close to 3′ end, ^1^ = mRNA degradation is a crucial mechanism of action of miRNA, other mechanisms, like translational repression or even translational activation, are also possible; abbreviations: RISC = RNA-induced silencing complex, TF = transcription factors, PRC2 = polycomb repressive complex 2, a complex with histone methyltransferase activity.

The entire human genome (in haploid dimension) is composed of approximately 3.2 billion of base pairs (bp), and only 30 million (ca. 1%) of bp are exonic sequences (i.e., genes) which encode mRNA particles translated to proteins [1,2]. In the past, only genes were considered important part of the DNA, whereas DNA elements other than genes were considered useless and called ‘junk DNA’. Today, it is known that these DNA fragments play an important role in regulation of the genes expression at different levels, i.e., chromatin organization and transcription factors binding, as well as direct influence upon mRNA transcripts and the process of their translation [3]. Non-coding fragments of the DNA are divided in untranscribed regions (55% of the whole human genome) and regions that are transcribed into so-called ‘non-coding RNAs’ (or ‘ncRNAs’, 43% of the whole human genome), which directly interfere with the process of transcription and translation [4]. Importantly, non-coding RNAs compose a significant part of the entire transcriptome (one-third in mice study by Okazaki et al.) [5]. The ncRNAs are divided into long non-coding RNAs (lncRNAs) and short non-coding RNAs, with the microRNAs (miRNAs or miRs) as, currently, the best known group. These two groups differ from each other in the number of forming nucleotides with 200 nucleotides as a categorizing number [6]. According to terms proposed by Fabbri et al., ncRNAs perform their regulatory effects through ‘interactor elements’ (IE) with the assistance of ‘structural elements’ (SE). Interactor elements are responsible for direct interactions between the ncRNA particle and other particles (nucleic acids, proteins, or lipids), whereas structural elements cause alterations in secondary and tertiary structures of the ncRNA particle affecting its reactivity [7]. The miRNAs regulate genes expression via direct binding to target mRNA particles through complementarity of nucleotide sequences and their degradation catalyzed by RISC complex, whereas lncRNAs mechanisms of action are more sophisticated, in recruitment of chromatin modifiers (like histone deacetylases and histone methyltransferases), regulation of protein activity, or regulation of miRNA availability by sponging mechanisms [8,9]. Mechanisms of action of miRNA and lncRNA have been briefly presented in Figure 1.

There are also ncRNA particles other than miRNA, such as circular RNA (circRNA), endo-siRNA (small interfering RNA), PIWI-interacting RNA (piRNA), small nucleolar RNA (snoRNA), tRNA-derived small RNA (tsRNA), and natural antisense transcripts (NATs) [7]. Although they are supposed to be negative regulators of other RNAs (for instance, circRNA acts as sponge which binds and inactivates miRNA particles and piRNA silences transportable genetic elements, transposons, and protects genome integrity), many aspects of their functions still remain unrevealed [10,11]. The non-coding RNA regulates different molecular pathways, including pathways involved in pathophysiological processes, like atherosclerotic plaque development and myocardial infarction [12]. In our review, we concentrate upon possible therapeutic applications targeted at ncRNAs in the process of atherosclerotic plaque development, myocardial necrosis, and cardiac remodeling after myocardial infarction. Moreover, preclinical studies in which agents targeted at ncRNAs involved in the aforementioned processes were used are described in this paper. Last but not least, limitations and challenges of these approaches are also discussed.

## 2. Agents Targeted at ncRNAs

Before reviewing the preclinical studies upon therapies targeted at ncRNA, it is necessary to present the agents used in these approaches. These agents are classified as inhibitors and agonists of either miRNAs or lncRNAs. The inhibitors are composed of two groups; the first group is antisense oligonucleotide (ASO), single stranded RNA (or DNA) particle perfectly complementary to targeted sequence which blocks its activity. Because RNA particles are chemically unstable, nucleotides constructing the ASO are chemically modified, which makes them more resistant to endonucleases, or endogenous degrading enzymes [13,14]. Moreover, chemical modifications also improve the ASO uptake by target cells. These chemical modifications include ribose modifications—2′-O-methyl (2′OMe), 2′-O-methyoxyethyl (2′MOE), 2′-F (fluoro), and LNA (locked nucleic acid-ribose chain chemically locked by -CH2-bridge connecting 2′-O and 4′-C in ribose), as well as phosphate modifications, including phosphorothioate (PS) bonds. In general, the ribose modifications increase ASO uptake, whereas PS bonds increase their resistance to endonucleases. Importantly, the term ’antagomiR’ means specific subtype of ASO with asymmetrical, phosphorothioate- and 2′-O-Me-modified, fully complementary oligonucleotides to the cognate miRNA sequence. A ‘gapmer’, that is a specific ASO with central DNA ‘gap region’ which binds target lncRNA sequence and induces its degradation through enzyme RNase H, is preferentially used for nuclear lncRNA degradation [15]. The second group of inhibitors are small interfering RNAs (siRNAs)—short double-stranded RNA particles which induce target miRNA or lncRNA degradation mediated through RISC complex [16]. In contrast, agents used as agonists of miRNA are chemically modified double-stranded nucleotides that resemble the precursor miRNA. Agonism of lncRNA is more difficult due to its size; therefore, either special adenoviral vectors (AAV) or induction of endogenous lncRNA by CRISPR/Cas9 gene editing are possible methods for restoring the function of endogenous lncRNA [17,18,19]. The agents used in ncRNA therapeutics are briefly presented in Table 1.

## 3. The ncRNA as Potential Targets in Therapy of Atherosclerosis: Experience from Preclinical Studies

### 3.1. Targeting ncRNA in Prevention of Myocardial Infarction—Atherosclerotic Plaque Progression

Myocardial infarction, in vast majority of cases, is a consequence of atherosclerotic plaque rupture or erosion [23]. Atherosclerotic plaque is a structure localized within the intima of artery (especially coronary artery) which develops gradually and in which progression is divided into a few stages. Briefly, a low-density lipoprotein (LDL) particle, after oxidation to oxidized LDL (oxLDL), crosses the barrier of endothelial cells and localizes within the intima. Then, these oxLDLs are phagocyted by macrophages, which initiate cytokines, chemokines, and local factors synthesis (especially M1, i.e., proinflammatory macrophages). The inflammation within the plaque is begun. Chemokines cause infiltration of other immune cells into the plaque (especially neutrophils and T cells). In addition, vascular smooth muscle cells change their phenotype from contractile to synthetic and migrate to the intima, creating scaffold for fibrous cap construction and actively participating in extracellular matrix remodeling (by altering collagens and proteoglycans structure) [24,25,26]. Meanwhile, macrophages become foam cells due to lipoprotein phagocytosis and undergo cellular death process (apoptosis and also necrosis in advanced plaques) [27]. Today, the inflammation and local immune response is considered as a crucial element in atherogenesis [28]. In one group of plaques, the inflammation is maintained at low-grade level (by anti-inflammatory mechanisms guided, e.g., by M2 macrophages), extracellular matrix is well-formed, and the entire structure is covered by a thick fibrous cap; these plaques are stable ones. However, in another group of plaques, local inflammation and immune cells infiltration are enhanced and damage caused by them results in increased necrosis within the plaque, extracellular matrix protein fragmentation, and fibrous cap thinning; these plaques are unstable (or vulnerable) ones, which makes them prone to be ruptured by blood stream [25]. On a ruptured plaque, thrombotic reaction is quickly initiated, and generated thrombus significantly obstructs coronary artery, leading to myocardial ischemia (or even cellular death—myocardial infarction) [25]. Moreover, atherogenesis, especially in initial stages, depends not only on oxLDL but also on other factors, like disturbed flow, which generates pro-atherogenic low-oscillatory shear stress [29]. Low oscillatory shear stress enhances the activity of pro-inflammatory nuclear factor k B (NF-kB), in contrast to high laminar shear stress, an inductor of atheroprotective Kruppel-like factor 2 (KLF2) [30]. All these aforementioned stages of atherosclerotic plaque generation, progression, and, finally, destabilization are regulated also by ncRNA particles. The miRNA and lncRNA, together with proteins regulated by them, compose complex networks [31,32]. A brief review of these network has been excellently provided by Fasolo for miRNA and circRNA [33], as well as by Pierce and Feinberg for lncRNA [34]. In Table 2 and Table 3, the examples of ncRNA (miRNA in Table 2, lncRNA in Table 3) and their influence upon atherogenesis are presented.

**Figure 2:**
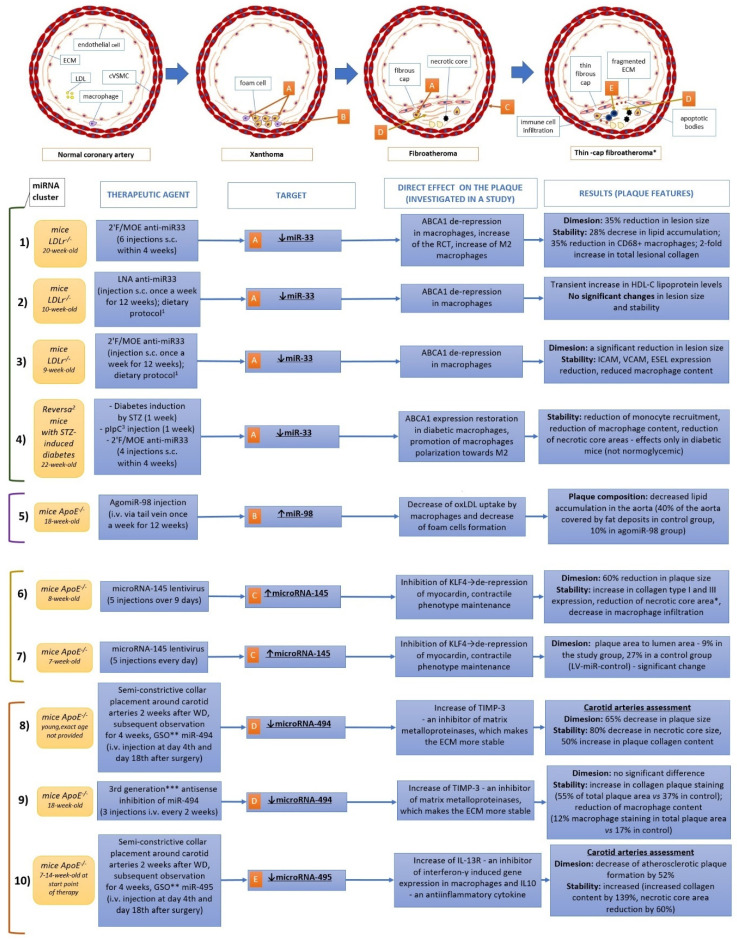
Examples of therapies targeted at ncRNA. **Studies:** (**1**) target miR-33 [82]; (**2**) target miR-33 [83]; (**3**) target miR33 [84]; (**4**) target miR-33 [43]; (**5**) target miR-98 [45]; (**6**) target miR-145 [59]; (**7**) target miR-145 [85]; (**8**) target miR-494 [86]; (**9**) target miR-494 [87]; (**10**) target miR-495 [88]. **Numbers ^(in superscript)^**: **^1^** = different dietary protocols; in study (**2**), normal chow diet for 2 weeks and Western diet (21% of fat) for 10 weeks; in study (**3**), Western diet for 12 weeks; **^2^** = Reversa mice is a transgenic mice (*LdLr*^−/−^*Apob*^100/100^*Mttp*^fl/fl^*Mx1*-*Cre*^+/+^), in which hypercholesterolemia can be reversed by Cre induction and subsequent *Mttp* gene inactivation [89]; **^3^** = pipC, polyinosinic polycytidylic RNA (pIpC), an agent used for Cre induction in Reversa mice model [90]; **Asterisks**: * = exact percentual changes not provided in the manuscript; ** = GSO, gene silencing oligonucleotide (synonym of ASO); *** = 3rd generation ASO are ASO modified with LNA (locked nucleic acids), PNA (peptic nucleic acids), or morpholinophosphoroamidate (MF) [91]. Abbreviations: 2′F/MOE = 2′ fluoro/methoxyethyl–modified, WD = Western diet (enriched with fat = 21%).

It is necessary to mention that many details about ncRNA (especially lncRNA and circRNA) mechanisms of action on cellular pathways involved in atherogenesis are still unknown. Noteworthy is that some studies provide different results upon the same microRNA particle and its impact on atherosclerotic plaque progression, suggesting ambiguous role of this miRNA in atherogenesis. For instance, miR-24 mimic, when transfected specifically to macrophages through rabies virus glycoprotein, RVG-9dR transfection system in ApoE^−/−^ mice after 12 weeks of atherogenic high fat diet, performs atheroprotective activity through MMP-14 down-regulation [54]. In contrast, when the miR-24 agomir dissolved in saline is administered to 8-week-old ApoE^−/−^ mice, its activity is performed mainly by SR-BI inhibition in liver and subsequent decrease of HDL uptake and reverse cholesterol transport, which results in increased atherosclerotic plaque [55]. In reference to potential therapies targeted at ncRNA, it needs to be revealed which pathways are crucial for atherosclerotic plaque progression, which pathways are only accessory and to what extent the pathways are regulated by systems of internal negative and positive feedbacks. Despite the fact that the solution of these problems is still fairly known, some investigations upon therapeutic agents targeted at ncRNA particles have been undertaken in animal models. Figure 2 summarizes the miRNA studies with agents targeted at miR-33, miR-98, miR-145, and miR-494/miR-495 (the last two microRNA derive from the same cluster 14q32 microRNA). These microRNA particles are considered to be regulators of crucial pathways involved in atherosclerotic plaque progression, and miR-33, miR-98, and miR-145 have been widely investigated in this field. Horie et al., in a study from 2012 upon double knock-out mice, has proven that miR-33 absence reduces the progression of atherosclerotic plaque [76]. Now, it is considered that therapy antagonizing miR-33 restores defective autophagy and apoptotic cell clearance through efferocytosis, reduces necrotic cores, and promotes macrophages differentiation towards anti-inflammatory M2 subpopulation [44,77]. The miR-98 targets and down-regulates LOX-1, a crucial element in atherosclerotic plaque initiation and progression which participates in oxLDL uptake by macrophages and foam cells generation [78]. The miR-145 is overexpressed in atherosclerotic plaque from hypertensive patients, and its agonizing therapy stabilizes vascular smooth muscle cells and maintains its physiological contractile phenotype [79,80]. Although there are a small number of studies upon miR-494/miR-495, it was demonstrated that miR-494 expression is increased two-fold in patients with significant (more than 70%) coronary artery stenosis [81].

Studies upon miR-33 presented on the Figure 2 underline the impact of experimental conditions upon final results, even if the same microRNA is investigated. Rayner et al. investigated miR-33 antagonizing therapy on atherosclerosis regression model in mice (older animals, 4-week duration of therapy) and obtained plaque regression by promotion of reverse cholesterol transport, whereas long-time therapy (12 weeks) by anti-miR-33 introduced in younger animals did not provide significant plaque change and did not affect reverse cholesterol transport, according to Marquat et al. [82,83]. Importantly, there is a limitation of studies upon miR-33 in rodents and their translation to humans because humans (unlike rodents) possess two *miR-33* genes (i.e., *miR-33a* and *miR-33b*) [92]. Noteworthy is that Figure 2 presents only therapies targeted microRNA in which agonists or antagonists of certain microRNAs were used and in which atherosclerotic plaque dimension and stability were evaluated. Therefore, studies on transgenic mice (double knock-out mice) were excluded because such therapy is not applicable in humans. However, other investigations on agonists and antagonists of different microRNAs, which indirectly regulate atherosclerotic plaque progression, were also undertaken. Agonizing of miR-335 (down-regulator of JAG-1 and inhibitor of pro-inflammatory Notch signaling pathway), miR-520c (down-regulator of RelA/p65, a subunit of pro-inflammatory NF-kB transcription factor), and let-7 g (down-regulator of platelet-derived growth factor (PDGF), mitogen-activated protein kinase kinase 1 (MEKK1), and also LOX-1) resulted in atherosclerotic plaque reduction [93,94,95,96]. Similar effects upon atherosclerotic plaque were observed when miR-135b (down-regulator of erythropoietin receptor) and miR-23a (down-regulator of ABCA1/G1) were antagonized by inhibitors [97,98]. The sustained miR-29 inhibition via LNA-miR-29 up-regulates extracellular matrix collagens, Col1A and Col3A (miR-29 target genes), and stabilizes the plaque, whereas miR-210 mimic prevents from carotid plaque rupture through APC down-regulation, subsequent β-catenin pathway enhancement and protection form α-smooth muscle cell apoptosis within the fibrous cap [99,100]. Interestingly, miR-181b modulation impacts atherosclerotic plaque in different ways. On one hand, therapy with miR-181b agomir reduces atherosclerotic plaque vulnerability through Notch-1 down-regulation in 8-week old ApoE^−/−^ mice [62]. On the other hand, miR-181b inhibition by locked nucleic acid resulted in decreased aneurysm formation and stabilized aneurysms in ApoE^−/−^ mice infused with angiotensin II [61].

The lncRNA also contributes to atherosclerotic plaque development, and the examples of lncRNA particles involved in this process are presented in Table 3. However, the majority of studies upon these particles were conducted on double knock-out animals (for example, ApoE^−/−^ MALAT1^−/−^). Therapy aiming to agonize or antagonize lncRNA with the use of mimicking or inhibiting agents is more difficult to perform, and exploration of the mechanisms of action is a great challenge. Nevertheless, investigations in this new area of research are being conducted. A study by Wu et al. revealed that knock-down of LincRNA-p21 by local injection of lentivirus-siRNA-LincRNA-p21 results in increased proliferation and increased neointima formation around injured carotid arteries. This study suggested an impact of LincRNA-p21 on the process of atherogenesis and explored a potential mechanism, i.e., binding to MDM2 protein, which leads to p53 release [101]. Sun et al. proposed a therapeutic approach towards atherosclerosis targeted at lncRNA particle RAPIA, expressed particularly in the late stage of atherosclerosis in macrophages. This study showed that injection of sh-RAPIA (a short hairpin RAPIA down-regulator) carried by adenoviral AAV2/9 vectors to 24-week-old ApoE^−/−^ mice (fed by atherogenic high fat diet for 16 weeks) resulted in reduction of lipid and macrophage accumulation, decrease of plaque size, and increase of collagen content. Moreover, it was demonstrated that RAPIA activity is at least in part mediated by miR-183 inhibition (by sponging) and subsequent increase of intraplaque macrophages proliferation. Importantly, atheroprotective effect of RAPIA silencing was similar to the effect induced by atorvastatin and combined therapy with the use of both sh-RAPIA and atorvastatin did not produce a stronger atheroprotective effect than either therapy alone [102].

### 3.2. Targeting ncRNA Regulating the Process of Myocardial Infarction

In the previous section, the regulation of the ncRNA involved in the process of atherosclerotic plaque progression and destabilization has been analyzed. However, when atherosclerotic plaque is ruptured, thrombus is generated, and the coronary artery is suddenly occluded, the myocardial tissue undergoes ischemia, leading to cellular death and myocardial infarction, which subsequently causes deterioration of heart physiological activity [103]. Apoptosis and necrosis of cardiomyocytes, consequent loss of contractile tissue and hemodynamic force, during myocardial infarction, induce processes of heart tissue wound healing, which, however, result in adverse cardiac remodeling. The remodeling may cause sustained impairment of ventricular function and heart failure development. Therefore, new treatment methods are needed to decrease morbidity and mortality, targeted both at processes of myocardial cellular death during the acute phase of myocardial infarction and the process of adverse cardiac remodeling [104,105]. The aforementioned processes are regulated also by ncRNAs (described well in the review by Das et al.), which might be a promising targets in future pharmacotherapy [106].

#### 3.2.1. Targeting the Process of Cardiomyocytes Cell Death during Acute Phase of Myocardial Infarction

The therapy targeted at the ncRNA particles involved in the processes of cellular death during myocardial infarction might be beneficial in reduction of the infarct size [107]. It has been evidenced that ncRNA reduces various types of cardiomyocytes cell death in hypoxic states, including necrosis, apoptosis, necroptosis, and pyroptosis, as described by D’Arcy [108]. Necrosis is an uncontrolled process of cell death which causes disruption of the cell membrane, leakage of the cytoplasmatic contents into the extracellular space, and induction of inflammatory response. Apoptosis is a form of programmed cell death, in which initiation depends on recruitment of caspases, leading to fragmentation of cellular proteins and DNA and subsequent formation of apoptotic bodies which, therefore, can be phagocytosed by neighboring cells. Necroptosis is a form of programmed necrosis regulated by receptor-interacting proteins 1 (RIP1) and 3 (RIP3), which leads to formation of necrosome and induces MLKL-dependent cell membrane permeabilization with subsequent necrosis of the cell. Last, but not least, pyroptosis is also a programmed cell death mechanism associated with inflammasome production, activation of inflammatory caspases, and formation of pores in cell membrane with cell swelling and release of intracellular contents into the surrounding tissues [109]. These cell death mechanisms are unfavorable in context of myocardial infarction, and interventions aimed to attenuate them might be efficient in reduction of infarction size.

One of such interventions targeted at ncRNA is enhancement of miR-133a activity. Li et al. revealed that, in hypoxic H9c2 cells (cell line from rat embryonic ventricular cardiomyocytes), overexpression of miR-133a enhanced proliferation and decreased number of apoptotic cells by 4.5% in comparison to the control cells. In contrast, silencing of miR-133a expression by transfecting lentiviral vectors with miR-133a inhibitor into these cells resulted in 5.5% increase of apoptotic cells when compared with the control cell group [110]. Moreover, Zhang et al. demonstrated that, in a C57/BL6 mouse model of myocardial infarction (MI) induced by ligation of coronary artery, overexpression of miRNA-133 by transfecting the adenoviral vector containing miRNA-133 results not only in higher values of left ventricular ejection fraction and fractional shortening but also in smaller area of myocardial necrosis in comparison to the controls [111]. Another protective ncRNA are miR-19a/19b (members of miR-17-92 cluster). It has been shown that direct injection of miR-19a or miR-19b mimics at heart regions surrounding the coronary artery ligation site resulted in preserved fractional shortening, reduction of scar size at 2 months following MI, and decrease in number of apoptotic cells [112]. Similarly, miR-494 also leads to protection from myocardial injury during ischemia, as its silencing significantly aggravated the injury, even though this miRNA targets both pro-apoptotic proteins, like PTEN, CaMKIIδ, and anti-apoptotic, like LIF [113]. On the other hand, miR-124 is a particle that increases cardiomyocyte apoptosis. It has been proven that, in a C57BL/6 mouse model, intramyocardial injection of antagomiR-124 immediately after coronary artery ligation results in decreased infarct area when compared to controls [114]. Additionally, in a same model of the MI in mice, microinjection of agomiR-325-3p reduced infarct size and ameliorated left ventricular parameters (increase of left ventricular ejection fraction, left ventricular fraction shortening, decrease of end-diastolic diameter and end-systolic diameter) by down-regulation of proteins involved in necroptosis, including RIPK1, RIPK3, and p-MLKL [115].

The loss of cardiomyocytes by apoptosis continues even long after the infarction [116]. There is a group of proapoptotic miRNA molecules up-regulated after myocardial infarction, for instance, miR-15, which was demonstrated in a porcine model. The miR-15 down-regulation prevents hypoxia-induced cardiomyocytes death, at least partially, due to increase of antiapoptotic proteins—Bcl2 and Arl2 (target mRNAs of miR-15). As a result, reduced cardiac remodeling and enhanced cardiac function measured as significant attenuation of fibrosis, improvement in ejection fraction, and decrease in LV volumes were observed 2 weeks after infarction in pigs treated with locked nucleic acid (LNA)-modified anti-miR-15 [117]. Other miRNA particles are also up-regulated in response to myocardial ischemia, like miR-199a, miR-210, or miR-34, and others, like miR-24, are down-regulated [118,119,120,121]. Interestingly, while miR-199a-5p presents cytotoxic activity through down-regulation of HIF-1α expression and subsequent cytoprotective p-GSK3β inhibition, its antisense strand—miRNA-199a-3p—promotes cell proliferation [122,123,124]. The miR-210 is also an inhibitor of apoptosis, which protects myocardial cell damage during oxygen-glucose deprivation/reperfusion therapy through down-regulation of E2F3 transcription factor, a cell cycle and apoptosis regulator. In addition, miR-34 is also a miRNA particle up-regulated in the heart in response to stress. Bernardo et al. showed in a mice model that LNA-anti-miR-34 administration attenuated pathological left ventricular remodeling in post-infarction hearts in part by increase of miR-34 target genes expression—*Sirt1*, *Notch1*, and *Pofut1*, which are responsible for cell survival, cardiac repair, and regeneration. The study group receiving LNA-anti-miR-34 for 8 weeks after myocardial infarction presented less decrease in fractional shortening (27 ± 2% comparing to 20 ± 1% in control group) and thicker left ventricle posterior wall, 0.71 ± 0.03 mm (comparing to 0.61 ± 0.02 mm in control group) [119]. In contrast, miR-24 is a cardioprotective miRNA, and its post-infarction delivery (through lipofectamine-mediated transfection in-vivo) resulted in decreased level of proapoptotic protein Bim, attenuated infarct size, and reduced cardiac dysfunction in mice, which was noted in magnetic resonance imaging as a higher ejection fraction (26.12 ± 2.58%, compared to 17.85 ± 2.45% in control group) and improved cardiac output (18.41 ± 2.14 mL/min in miR-24-treated group and 12.15 ± 0.97 mL/min in control group) [120].

Although principal mechanisms of cellular death within heart muscle during myocardial infarction are aforementioned apoptosis, necrosis, necroptosis, and pyroptosis, there is another mechanism—autophagy—, which causes autophagocytosis and degradation of unnecessary proteins, organelles, and cellular compartments. This process plays a protective role during myocardial infarction [125]. Beneficial effects of autophagy activation and reduction of cardiomyocytes apoptosis are induced by overexpression of some miRNA, for example, miR-99a. Intramyocardial injection of lentivirus-mediated miR-99a attenuated pathological remodeling, as well as improved survival rate and cardiac function, in mice after MI, probably via an mTOR/P70/S6K signaling pathway. Four weeks after MI, the ejection fraction was 48.11 ± 1.14% and 39.61 ± 1.31% in the lentivirus-mediated miR-99a-treated group and control group, respectively, whereas fractional shortening amounted to 23.90 ± 0.62% in the study group of C57/BL6 mice and to 19.32 ± 0.71% in control group. Changes in both ejection fraction and fractional shortening were statistically significant [126]. Similar results have been achieved in older mice by inhibition of miR-22 by LNA-based anti-miR approach, which caused activation of cardiac autophagy and resulted in cardiac function improvement and less decreased ejection fraction in comparison with animals from control group [127].

Apart from miRNA, the studies investigating lncRNA and circRNA were also conducted. For instance, it has been revealed that injection of human mesenchymal stem cell-derived exosomes transfected with lncRNA KLF-3-AS1 into rats with MI (induced by ligation of coronary artery) resulted in reduction of MI area and decrease of apoptosis rate and pyroptosis-related proteins expression (such as NLRP3, ASC, and caspase-1), as well as inflammatory cytokines (IL-1β and IL-18). These effects were exerted through sponge effect on miR-138-5p and down-regulation of miR-138-5p/Sirt1 axis [109]. In reference to circRNA, Cai et al. revealed that, in hypoxic neonatal rat ventricular myocytes, plasmids-mediated overexpression of circ-Ttc3 reduced expression of cleaved-PARP and caspase-3, which decreased number of apoptotic cells in comparison to control cells through sponging of miR-15b-5p [128]. Moreover, direct intramyocardial injection of viral particles expressing circFndc3b (in mouse model of coronary artery-ligation-induced MI) results in its overexpression, leading to significant improvement in ejection fraction, fractional shortening, restoration of left ventricular dimension, and reduction of cardiomyocytes apoptosis when compared to control animals [129].

#### 3.2.2. Targeting ncRNA in Prevention of Unfavorable Cardiac Remodeling after Myocardial Infarction

The consequence of myocardial cell death are tissue fibrosis and subsequent cardiac remodeling. This process depends not only on cellular death and its consequences (mentioned in the previous section) but also on extracellular matrix structural alterations leading to fibrosis, cardiac muscle hypertrophy, and neovascularization, which are controlled by inflammation within post-infarcted myocardium [8]. Importantly, although cardiac hypertrophy is an adaptive response, hypertrophic myocardial tissue is dysfunctional due to cellular damage and hypoxia related to capillary rarefaction [130,131,132]. Animal studies proved that modulation of ncRNA involved in these processes improves cardiac function and attenuates adverse remodeling, assessed by heart size, shape, mass, and ejection fraction, and end-diastolic and end-systolic volumes, as well as peak force of contraction [133,134].

During extracellular matrix restructuration after myocardial infarction, the role of miR-21 is particularly important. On one hand, this micro-RNA promotes fibroblasts survival and interstitial fibrosis through inhibition of sprouty homologue 1 (Spry1) and subsequent activation of ERK-MAP kinase signaling, as well as by targeting Smad7, a negative regulator of pro-fibrotic transforming growth factor beta (TGFβ) [135,136,137]. On the other hand, miR-21 decreases level inflammatory cytokines and CD11b+ monocytes/macrophages infiltration within cardiac tissue. This anti-inflammatory effect is caused by targeting and degradation of kelch repeat and BTB (POZ) domain containing 7 (KBTBD7), which subsequently results in decreased p38 and NF-kB signaling. Importantly, miR-21 knockout mice presents significantly increased infarct and scar size [138]. Another miRNA regulating cardiac remodeling is miR-17a-3p, which stimulates cardiomyocyte proliferation and promotes physiological (exercise-induced) hypertrophy through TIMP-3 down-regulation and subsequent de-repression of EGFR/JNK/SP-1 signaling pathway. Moreover, treatment with miR-17a-3p agomir (therapy initiated 24 h after reperfusion) resulted in preservation of cardiac function (fractional shortening and ejection fraction) [139]. A micro-RNA particle which directly targets collagen synthesis and subsequently affects extracellular matrix structure is miR-590-3p. This miRNA inhibits fibroblasts cell proliferation, migration activity, and collagen components (Col1A1 and Col3A) synthesis through the down-regulation of zinc finger E-box binding homeobox 1 (ZEB1), which was shown in the study upon human cell fibroblasts (HCF) [140]. This study also confirmed significantly lower miR-590-3p expression and higher expression of ZEB1 mRNA in minipigs with myocardial infarction comparing to controls without MI.

Angiogenesis is a factor that preserves form adverse cardiac remodeling after myocardial infarction [141]. This process is also regulated by microRNA particles, like miR-34a, miR-26a, and miR-378. In a study by Boon et al., LNA-based therapy with anti-miR-34a, which increases anti-apoptotic *PNUTS* expression (a target gene of miR-34a) within the cardiac muscle, resulted not only in cell death reduction but also in increased capillary density in border zone of infarcted area [142]. Similarly, inhibition of miR-26a in mice by LNA resulted in induction of myocardial angiogenesis measured by CD31 and isolectin staining, which was associated with significantly improved LV ejection fraction (21% after 48 h and 32% after 8 days) [143]. MiR-378 is an important regulator of pro-angiogenic activity of CD34+ progenitor cells. The expression of this miRNA is increased in CD34+ progenitor cells isolated from patients with myocardial infarction with ST elevation (STEMI) in comparison with stable coronary artery disease patients and healthy subjects, which might contribute to increased tissue repair program activated in the infarcted area of the heart [144].

The process of cardiac remodeling is regulated not only by miRNA but also by lncRNA and circRNA particles. An important mediator of TGF-β pathway is a lncRNA particle called *Safe*, and its knock-down by lentivirus significantly inhibited fibrosis, which resulted in improved ejection fraction and fractional shortening in mice after 28 days from the moment of MI induction by LAD ligation [145]. In addition, lncRNA called ‘lnc-Ang362′ targets and inhibits Smad7, a negative regulator of TGF-β and protector from myocardial fibrosis. The knock-down of lnc-Ang362 resulted in increased Smad7 expression and decreased collagen I/III synthesis [146]. Apart from TGF-β, a factor particularly involved in cardiac fibrosis is connective tissue growth factor (CTGF) [147,148,149]. Interestingly, CTGF pathway is under negative control of miR-30, which is sponged (and blocked) by lncRNA named n379519. Inhibition of n379519 caused decrease in α-SMA, collagen 3A1, collagen 8A1, and fibronectin, which resulted in attenuation of myocardial interstitial fibrosis, limitation of total collagen volume and prevention from LVEF reduction [150,151]. Another example of the role of sponge mechanism is lncRNA GAS5, which sponges miR-21. The knockdown of this lncRNA promoted myocardial cells survival [152].

Increasing evidences demonstrated that lncRNA might play a pivotal role in the process of cardiac hypertrophy. An example is cardiomyocyte regeneration-related lncRNA (CRRL), which is involved in negative regulation of cardiomyocytes proliferation and its knockdown attenuated post-MI remodeling and preserved cardiac function in rats. CRRL directly binds miR-199a-3p, a suppressor of *Hopx* gene. The HopX (homeodomain only protein X) drives cardiac hypertrophy by recruiting HDAC and up-regulating hypertrophic mediators, like ERK-1, ERK-2, or IGF-1 [153,154,155]. Similarly, cardiac muscle is protected by miR-489, which down-regulates the myeloid differentiation primary response gene (*Myd88*) and prevents from cardiac hypertrophy. This microRNA is blocked by cardiac hypertrophy related factor (CHRF), a lncRNA which acts as endogenous sponge for miR-489 [156]. The two aforementioned examples of lncRNA emphasizes the role of lncRNA-miRNA networks in the regulation cardiac hypertrophy. In addition, an example of protective ncRNA is miR-155, in which loss prevents the progress of heart failure and suppresses cardiac hypertrophy in mice model [157].

The circRNA proteins involved in the regulation of cardiac adverse remodeling are heart-related circRNA (HRCR) and circ-FOXO-3. The former circRNA inhibits cardiac hypertrophy by acting as endogenous sponge for miR-223, whereas the latter circRNA promotes cellular senescence and aggravates doxorubicin-induced cardiomyopathy [158,159].

#### 3.2.3. Preclinical Studies Examples

Therapies which targets ncRNA particles regulating the processes of myocardial cell death and adverse cardiac remodeling are under investigation in preclinical models. Some of these trials and their results have already been mentioned above, and the details of selected therapies aimed to prevent from negative cardiac remodeling and deterioration of heart function in animal models are presented in Table 4. Interestingly, there are investigations upon ncRNA that were conducted in specific conditions, like diabetes. For instance, miR-17 was up-regulated in diabetic mice (diabetes induced by streptozotocin), and its inhibition by antagomir significantly improved left ventricle function and decreased infarct size in this group of mice [160]. Apart from approaches targeted at one specific miRNA or lncRNA particle, the trials aiming to assess synergic effects of two ago- or antago-miRs have also been undergone. Combination of two agomiRs—agomiR-21 and agomiR-146a—caused more accentuated decrease of infarct size and preservation of ejection fraction than use of one of these agomiRs in mice model [161].

## 4. Challenges in Therapies Aimed to ncRNA

The therapies targeted at ncRNA are promising in the field of cardiovascular diseases; however, there are important challenges that need to be overcome. Up to date, a study upon miravirsen, which antagonizes miR-122, has been completed and has revealed its positive effect in patients with hepatitis C virus infection [168]. In the field of cardiology and nephrology, an ongoing clinical study HERA (randomized, double-blinded) on lademirsen, a miR-21 antagonist as a potential inhibitor of cardiac fibrosis, is being undertaken in patients with Alport syndrome (NCT02855268). It must be emphasized that therapies targeted at ncRNA and gene expression control machinery might provide long-lasting effects which are difficult to predict, and homogenous animal models are likely insufficient to profoundly investigate this matter. Another problem is administration of drugs targeted at ncRNA. In contrast to possibility of long-lasting effects provided by these drugs, therapeutic effects of some ASO could be too transient, and repeated administration would be necessary. Therefore, toxic effects of ncRNA-targeted drugs should also be considered [169]. These toxic effects include hybridization-dependent toxicity (involving off-target effects and potential mutagenesis due to unspecific binding to DNA) and hybridization-independent toxicity (involving liver cell damage and TLR-mediated inflammation enhancement) [17]. Last, but not least, ncRNA-targeted therapeutic agents should be delivered to the tissue of interest. In order to address the drug at selected site, special transport systems are constructed, like nanoparticles with ligands specific to target cell receptors or recombinant adeno-associated virus (AAV) with high affinity to cardiomyocytes [12,16]. In order to overcome the problems with site-specific delivery, very sophisticated methods are currently under investigation. An example is multifunctional biomimetic nanoparticle system (ternary polyplexes coated with ApoA-I resembling HDL particles), which not only interacts with specific receptors on macrophage, but also creates positive feedback loop which facilitates the drug delivery to the macrophage. Briefly, down-regulation of one receptor (SR-A on macrophages by siRNA released by the nanoparticle) results in enhancement of the other receptor activity (CD36), which furtherly promotes binding to macrophages and siRNA release into this cell [170]. Another method of drug delivery at specific site is a use of nanoplatforms targeted at different cells (like core-shell nanoparticles composed of PGLA core and three external layers: lipid layer, apoA-I layer for enhanced entry into macrophages, and hyaluronic acid layer for endothelial cell targeting) [171]. Such systems might increase specificity of drug release to the tissue of interest and assure that a higher percentage of drug dose will reach the destination cell and cause the effect. Advantages and disadvantages of different RNA-based therapeutic approaches (adenoviral vectors, lentiviral vectors, oligonucleotide-based therapy, exosome-based RNA therapy, and nanoparticle-based gene delivery) have been excellently presented by Lu and Thum [172]. The issue of potential mutagenesis has also been underlined; it is considered that oligonucleotide-based therapeutic agents do not pose a threat to genomic integrity, unlike usage of lentiviral vectors. Today, the new experience is being gained in the field of RNA-based therapies due to investigations upon vaccines against SARS-CoV2 which use either artificial nano-encapsulated mRNA strands or viral vectors [173].

## 5. Conclusions

The therapies targeted at ncRNA (microRNA, circRNA, and lncRNA) have a great potential in the field of cardiovascular diseases treatment. Blocking or mimicking specific ncRNA particles might result in effective inhibition of atherosclerotic plaque progression, limitation of myocardial necrosis, and prevention from unfavorable cardiac remodeling. However, due to significant challenges (especially possible long-lasting effects, which are difficult to predict in a heterogenic human population), it is still difficult to even design appropriate clinical study. Nevertheless, therapies targeted at ncRNA are hopeful strategies for future treatment of cardiovascular diseases.

## Figures and Tables

**Table 1:** Methods of therapeutics targeted at miRNA and lncRNA. Based upon References [14,17,20,21,22].

	Inhibition	Activation
miRNA	ASO—chemically modified single-stranded RNAs (ribose and phosphate modifications) siRNA (RNA interference)	AgomiRs, miRNA mimics—chemically modified double-stranded RNAs.
lncRNA	● ASO: ○ shRNA (short hairpin RNA) ○ ‘gapmer’ (central DNA ‘gap region’) ● siRNA (RNA interference)	lncRNA mimics lncRNA induction (CRISPR/Cas9 gene editing)

**Table 2:** The miRNAs and their impact upon atherogenesis. * indicates mRNAs (names in **bold**) which are the targets for miRNAs and their RISC-mediated degrading activity; ** in advanced lesions, pro-atherogenic factors are also considered as factors promoting atherosclerotic plaque vulnerability. Abbreviations: EC = endothelial cells, FAO = fatty acid oxidation. ^1^ = a study in which ApoE^−/−^ mice with specific knock-down of miR21 in bone-marrow cells were investigated; ^2^ = a study in which miR-21 knock-down Sprague Dawley (SD) rats were used; ^3^ = a study in which miR-24 was specifically transfected to macrophages; ^4^ = a study conducted on ApoE^−/−^ mice with knockout of both miR-143 and miR-145.

The microRNAs in the Process of Atherosclerotic Plaque Progression (Categorized by Stages)			
miRNA	Stage	Function	Mechanism, Target mRNA * and References
miR-30c	Initial—lipid metabolism	Atheroprotective	Down-regulation of **MTTP** *, an essential factor in lipidation of apoB [35]
miR-10a	Initial—EC	Atheroprotective	Down-regulation of **GATA6** *, an inducer of adhesion molecule VCAM-1 in the EC [36]
miR-92a	Initial—EC	Atherogenic	Down-regulation of **KLF2** * and **KLF4** * expression [37]
miR-34a	Initial—EC	Atherogenic	Down-regulation of **BCL2** * [38] (antiapoptotic mediator) and **SIRT1** * (a class III histone deacetylase, participating in maintenance of cellular longevity) [39,40]
miR-126	Initial—EC	Atheroprotective	Down-regulation of **VCAM-1** * (cell adhesion molecule for leukocytes) and **Dlk-1** * (a negative regulator of EC proliferation) [41]
miR-19	Progression—macrophages	Atherogenic	Down-regulation of **ABCA1** * (a transporter essential for reverse cholesterol transport through HDL) [42]
miR-33	Progression—macrophages	Atherogenic	Down-regulation of **ABCA1** * (a transporter essential for reverse cholesterol transport through HDL) [43] and **AMPK** *, a kinase involved in FAO activation and macrophages polarization towards anti-inflammatory M2 phenotype [44]
miR-98	Progression—macrophages	Atheroprotective	Down-regulation of **LOX1** *, a receptor for oxLDL participating in foam cell formation [45]
miR-155	Progression—macrophages	Atherogenic	Down-regulation of **BCL6** *, an antiapoptotic protein and inhibitor of NF-kB signaling [46,47], and **SHIP1** *, inositol phosphatase blocking PI3K pathways and modulating T-lymphocytes activity [46,48]
miR-182	Progression—macrophages	Atherogenic	Down-regulation of **HDAC9** *, which results in increase of lipoprotein lipase (LPL) expression in macrophages and increased uptake of lipoproteins [49]
miR-590	Progression—macrophages	Atheroprotective	Down-regulation of **LPL** *, which results in decreased lipoprotein uptake by macrophages [50]
miR-124	Advanced—VSMC, ECM	Atherogenic	Down-regulation of **P4HA1** *, a key enzyme in collagen synthesis [51]
miR-21	Advanced **—VSMC, ECM	Ambiguous	Atheroprotective: down-regulation **MKK3** * (leading to ablation of MKK3-p38-CHOP pro-apoptotic pathways) [52] ^1^ Atherogenic: down-regulation of **PTEN** * (leading to Akt/ERK signaling pathways activation and VSMC proliferation) [53] ^2^
miR-24	Advanced **—VSMC, ECM	Ambiguous	Atheroprotective: down-regulation of **MMP14** *, matrix metalloproteinase participating in extracellular matrix fragmentation [54] ^3^ Atherogenic: down-regulation of **SR-BI** * in liver, which causes inhibition of HDL uptake and reverse cholesterol transport [55]
miR-223	Advanced **—VSMC, ECM	Ambiguous	Inhibition of **IGF-1R** * in VSMC, which results in atheroprotection on earlier stages [56], but might participate in destabilization on advanced stages (before rupture) [57,58]
miR-143/145	Multi-stage	Ambiguous	Atheroprotective: down-regulation of **KLF4** *, resulting in increased myocardin expression and VSMC contractile phenotype maintenance [59] Atherogenic: down-regulation of **ABCA1** *, (a transporter essential for reverse cholesterol transport through HDL) [60] ^4^
miR-181b	Multi-stage	Ambiguous	Atherogenic: down-regulation of **TIMP3** *, an inhibitor of destabilizing metalloproteinases [61] Atheroprotective: down-regulation of **NOTCH1** *, a transmembrane protein promoting macrophage pro-inflammatory polarization [62]

**Table 3:** The lncRNAs and their impact upon atherogenesis; * in advanced lesions, pro-atherogenic factors are also considered as factors promoting atherosclerotic plaque vulnerability ^1^= spongue effect results in inhibition of trapped miRNA; ^2^ = atheroprotective activity of ANRIL might be associated rather with its circular form, circANRIL, according to Holdt et al. [63].

The lncRNA in the Process of Atherosclerotic Plaque Progression			
lncRNA	Stage	Function	Mechanism and References
SNHG12	Multi-stage	atheroprotective	Binding to DNA-PK, facilitating interaction with Ku70 and Ku80, resulting in appropriate response to DNA damage [64]
HOTAIR	Initial—EC	atheroprotective	Scaffold for PRC-2 and LDS-1 (histone modifications) [65], EC protection from senescence [66]
SENCR	Initial—EC	atheroprotective	Maintaining EC layer integrity by CKAP-4 binding in cytoplasm and prevention from VE-cadherin internalization induced by CKAP-4 [67]
NEXN-AS1	Initial—EC	atheroprotective	Binding the DNA region of *NEXN* promotor and enhancing its expression (NEXN = inhibitor of TLR4 oligomerization and NF-kB activity) [68]
MIAT	Progression—macrophages	atherogenic	Sponging effect ^1^ on miR-149-5p and prevention from CD47 degradation, leading to impaired efferocytosis (CD47 = efferocytosis inhibitor, ‘don’t eat me’ signal, a target for miR-149-5p) [69]
MeXis	Progression—macrophages	atheroprotective	Indirect promotion of ABCA1 expression (by guiding transcription factor DDX-17 to a nearby ABCA1 locus) [70]
NEAT1	Advanced—VSMC *	atherogenic	Inhibition of WDR-5 activity, a histone modifier that promotes contractile VSMC phenotype [71]
MALAT1	Multi-stage	atheroprotective	Autophagy activation in VSMC (sponging ^1^ of miR-142-3p, a down-regulator of ATG7, an autophagy-related, beneficial protein) [72], decrease of hematopoietic cells in bone marrow, decrease leukocyte adhesion to EC (partially through miR-503 inhibition) [73]
ANRIL	Multi-stage	ambiguous	**Atheroprotective** ^2^: up-regulation of CLIP-1, EZR, and LYVE-1, which promotes EC physiological functions (e.g., nourishment through micropinocytosis) [74] **Atherogenic**: high-risk alleles of ANRIL (SNP in chromosome 9p21.3) promoting linear ANRIL isoform associated with increased atherosclerosis [75]

**Table 4:** The exemplary therapies upon ncRNAs and their outcomes in animal studies. ^1^ = injections at day 0, 1, 3 and then every 3 days until 28th day after myocardial infarction, ^2^ = minicircles, products of site-specific intramolecular recombination driven by bacteriophage ΦC31 integrase, * = in these manuscripts, exact quantitative data were not provided.

ncRNA of Interest	ncRNA Mechanism of Action	Intervention	Model	Outcome	References
miR-144 (miRNA)	mTOR down-regulation → promotion of autophagy	**Enhancement** MiR-144 i.v. administration (day 0, 1, 3, and later) ^1^	C57BL/6 mice, MI by LAD ligation	Increased fractional shortening (approximately 2-fold) *, increased LVEF * Decreased scar length (approximately 2-fold) * Decreased cardiomyocyte size	[162,163]
miR-210 (miRNA)	Efna3 and Ptp1b (angiogenesis inhibitors) down-regulation	**Enhancement** Minicircle ^2^ DNA (containing miR-210 precursor) administration	Adult female FVB mice, MI by LAD ligation	Increased LVEF: 27.8% vs. 24.2% (after 8 weeks) Reduced apoptosis (TUNEL staining): 0.13% vs. 0.22% (after 8 weeks) Smaller infarct faction (26.5% vs. 35.4%)	[164]
miR-31 (miRNA)	Down-regulation of Tnnt2 (troponin T), Nr3c2, E2f6, and Timp4, factors responsible for cellular viability and ECM stability	**Inhibition** LNA-modified anti-miR injected s.c., after 2 and 16 days from MI	Male adult Wistar Rats, MI by LAD ligation	Absolute improvement in LVEF (10 p.p.) vs. absolute deterioration in LVEF (17 pp) in control group after 4 weeks Increased cardiac output Similar infarct size	[165]
miR-199a-3p	Entothelin-1 (ET-1) down-regulation	**Enhancement** miR-199a-3p mimics (lipofectamine RNAiMAX)	Female CD1 mice, MI by LAD ligation	Increased LVEF (37.5% vs. 24% in control group) Smaller infarct size (percentage of the left ventricle–18% vs. 28% in control group)	[166]
miR-590-3p	Collagen synthesis induction through ZEB1 down-regulation	**Enhancement** miR-590-3p mimics	Female CD1 mice, MI by LAD ligation	Increased LVEF (48.5% vs. 24% in control group) Smaller infarct size (percentage of the left ventricle–14% vs. 28% in control group)	[166]
circFndc3b (circRNA)	Interaction with FUS RNA-binding protein → VEGF-A up-regulation	**Enhancement** AAV-9 mediated overexpression	C57BL/6 male mice, MI by LAD ligation	Increased LVEF *, increased fractional shortening * Reduction of infarct size (approximately 2-fold) *	[129]
MEG3 (lncRNA)	Direct p53 binding and activation → promotion of ERS- and NF-kB mediated myocardial apoptosis	**Inhibition** si-MEG3 (small interfering RNA) in lentiviruses	C57BL/6 male mice, MI by LAD ligation	Lower degree of cardiac fibrosis (decreased collagen fraction) * Decreased infarct size * Less spherical shape of heart	[167]
